# In safe hands – patients with knee and hip osteoarthritis expectations and experience of physical therapist-led triage in a secondary care setting

**DOI:** 10.1186/s12891-026-10179-3

**Published:** 2026-07-07

**Authors:** Linnéa Gustavsson, Daniel Broman, Susanne Beischer, Karin Samsson

**Affiliations:** 1 Institute of Clinical Sciences, Department of Orthopedics, Sahlgrenska Academy, University of Gothenburg, Gothenburg, Sweden; 2 Sportrehab, Sports Medicine Clinic, Gothenburg, Sweden; 3 Institute of Neuroscience and Rehabilitation, Department of Health and Rehabilitation, Sahlgrenska Academy, University of Gothenburg, Gothenburg, Sweden; 4 Capio Ortho Center Gothenburg, Gothenburg, Sweden

**Keywords:** Physiotherapy-led triage, Expectations, Experiences, Osteoarthritis, Secondary care, Patient-centered care

## Abstract

**Background:**

Physical therapist-led (PT-led) orthopedic triage is a care model designed to improve access to specialist care for patients with musculoskeletal disorders (MSK), including osteoarthritis (OA). Results from a previous RCT comparing PT-led triage with standard care (orthopedic surgeon assessment) showed that patients perceived the received care to be of good quality, however also showed (i) lower levels of participation in decision-making and that (ii) their expectations on the assessment were fulfilled to a lesser extent. Therefore, the aim of this study was to explore the expectations of the assessment and experiences of patients with hip or knee OA regarding PT-led triage in an Swedish orthopedic clinic.

**Methods:**

An explorative qualitative research design with an inductive approach was used. Patients who had undergone PT-led triage for their hip/knee OA at a hospital based orthopedic clinic were invited to participate. A purposive sampling was used to get a variation in gender and assessment outcome. Semi-structured, one-to-one interviews were conducted in Swedish. The interviews were audio recorded, transcribed verbatim and analyzed with content analysis according to Graneheim and Lundman. Thereafter the results were translated into English.

**Result:**

Twelve patients were included in the study. The patients’ expectations and experiences about PT-led triage were classified into 2 themes; *In safe hands* and *A care model in progress*, with a total of 6 categories: Highly skilled and professional PT, Sense of involvement and empowerment, Feeling validated, An innovative and effective care model, Expectations on the meeting and Opportunities for Improvements.

**Conclusion:**

The main findings of this study are that patients felt they were meeting an expert, experienced a sense of involvement in their care, and viewed PT-led triage as a valuable model of care although some patients reported a lack of information regarding the care process. The results from this study of patients’ expectations and experiences may be used to further refine and improve this model of care, thereby supporting its implementation on a broader scale.

**Trial registration:**

The study was prospectively registered in Clinical Trials Gov NCT04665908, registered 07/12/2020.

**Supplementary Information:**

The online version contains supplementary material available at 10.1186/s12891-026-10179-3.

## Background

Osteoarthritis (OA) ranks among the leading causes of disabilities and overall burden worldwide [1]. The number of primary hip and knee replacements (THA/TKA) in 2023 increased by nearly 20% compared to the year before the pandemic [2]. The expanding patient population requiring assessment whether surgical intervention is appropriate has led to increased demand for primary care and orthopedic clinics [3–5]. For patients, this results in longer waiting times from referral to assessment at the orthopedic clinic [5, 6]. To improve accessibility to orthopedic clinics for patients with OA, access pathways need to be optimized [6, 7]. One approach to achieve this is through implementation of a physical therapy-led (PT-led) orthopedic triage care model. PT-led triage means that patients are assessed by a physical therapist (PT) to determine the most suitable treatment pathway, i.e. surgical intervention, initiated/continued rehabilitation in primary care or further management by the general practitioner (GP) [8, 9].

Previous research on PT-led triage for patients with musculoskeletal disorders (MSK) in primary care has shown that this model of care may increase surgery conversion rate and reduce waiting times [7, 10], while demonstrating no significant differences in patient-reported outcomes in either short- or long-term [11]. Furthermore, studies exploring patient satisfaction with PT-led triage for MSK suggests that this approach provides care that patients perceive as satisfactory [12, 13]. However, only a limited number of studies have examined patients’ expectations and experiences within PT-led triage for OA.

At Sahlgrenska University Hospital in Gothenburg, Sweden, a PT-led triage model for patients with hip or knee OA was introduced in 2017. Patients referred for an orthopedic assessment, who were informed that their initial evaluation would be conducted by a PT, were assessed to determine whether THA or TKA was appropriate. Patients received a preliminary decision regarding surgical possibilities during the PT-assessment. Final decisions were subsequently made at a weekly PT-OS conference, after which patients were notified of the outcome by mail. This model has since been evaluated in an RCT [14], which found patient-perceived quality of care to be equivalent between PT-led triage and standard care (assessment by an OS). However, the RCT also reported significant differences where patients in the PT-led triage group perceived that they received more information about how to manage their OA, whereas those assessed in standard care felt more involved in decision-making and that their expectations for the appointment were met at a higher degree.

Patient involvement is a cornerstone of patient-centered care (PCC), underscoring the importance of understanding how patients experience engagement in care-related decisions within various triage models [15, 16]. To further understand how PT-led triage meets patients’ needs and expectations, additional exploration of patient’s perspective is warranted. Therefore, the aim of the present study was to investigate the expectations of the assessment and experiences of patients with hip and knee OA regarding the PT-led triage model implemented at Sahlgrenska university Hospital.

## Method

### Study design

An explorative qualitative research design with an inductive approach was chosen for this study. The Consolidated Criteria for Reporting Qualitative Research (COREQ) checklist [17] was used in planning and reporting the result of the study. In accordance with the Declaration of Helsinki ethical approval was obtained (registration number 2024-01787-01) from the Swedish Ethical Review Authority prior to the initiation of the study.

### Study setting and participants

Swedish speaking patients who were referred for orthopaedic assessment were informed about the new procedure with the care model PT-led triage and were assessed by a PT for their hip or knee OA at the orthopedic clinic at Sahlgrenska University Hospital, Gothenburg, Sweden between September and November 2024 were invited to participate in the study. The first author received a weekly list of assessed patients from the assessing PTs, who were aware of the ongoing study. A purposive sampling approach was used to get a variation in gender and assessment outcome (i.e. surgical intervention vs. no surgical intervention). The number of included patients was set to 10–15, based on previous qualitative studies, to ensure variation and achieve data saturation [12, 18, 19]. Patients were contacted by phone by the first author within one week after their assessment. Upon agreeing to participate, the time and location for the interview were scheduled. All patients provided written informed consent prior to the interview. The interviewer had no other prior contact with any of the patients before the study.

### Data collection

Semi-structured one-to-one interviews were conducted by the first author who has post graduate training in qualitative research methods and 10 years of clinical experience working with patients with OA. To avoid influencing the patients’ responses, the interviewer introduced herself as a researcher. The location of the interviews was chosen by the patients; eight were conducted via video conference, two at rehabilitation clinic (one at SBs and one at KS workplace), one at the research center and one by phone. The interviews were conducted in Swedish, lasted between 14 and 40 min (mean duration: 25 min), were audio recorded and later transcribed verbatim. Field notes were also taken during the interviews. To ensure that all relevant topics were covered, an interview guide was developed (Supplementary file), focusing on questions related to the patients’ expectations and experiences of the PT-led triage assessment, as well as their general views on the care model. The guide was developed based on findings from the previously published study about patient perception of quality of care by Gustavsson et al. [14] and was tested in two pilot interviews. No changes were made to the interview guide following the pilot testing.

### Data analysis

To identify patterns and gain a deeper understanding of the patients’ expectations and experiences about PT-led triage at an orthopedic clinic, a qualitative content analysis was conducted following the approach described by Graneheim and Lundman [20].

The transcribed interviews were initially read multiple times to gain a sense of the whole and an overall understanding. The first step of the analysis involved identifying meaning units related to the aim of the study. In the next step, the meaning units were coded, i.e. labeled in a way that preserved their meaning and contextual relevance. The first two interviews were read and meaning units identified as well as labeled by all the three authors independently and thereafter followed by discussion and consensus before the two of the authors (LG, DB) continued with the rest of the interviews. Based on similarities and differences, the codes were then organized into subcategories, categories and finally, overarching themes. Each step of the analysis was conducted independently by two authors (LG, DB) followed by discussion and consensus before proceeding to the next step. The senior author (KS), who has prior experience in qualitative research, also participated in the discussion at each stage of the analysis. The language translation from Swedish to English was made after completing the analysis. All authors involved in the analysis are registered PTs working at primary care rehabilitation clinics.

## Results

Thirty-one patients were eligible for inclusion, of whom eleven could not be reached. Of the 20 patients invited to participate, 12 patients (6 men, 6 women) agreed participation, including the two pilot interviews, which were included in the analysis. Patients’ characteristics are presented in Table 1.

**Table 1:** Characteristics of study participants

Participant no	Gender (M/F)	Age	Level of education	Affected joint	Assessment outcome	Informed letter
1	M	71	University	Knee	?	No
2	M	76	University	Hip and knee	Surgical treatment appropriate	Yes
3	F	71	University	Knee	Non-surgical treatment	Yes
4	M	62	University	Knee	Non-surgical treatment	No
5	F	79	University	Knee	Surgical treatment appropriate	Yes
6	M	72	High school	Hip and knee	Surgical treatment appropriate	Yes
7	M	73	High school	Knee	Surgical treatment appropriate	Yes
8	M	69	High school	Knee	Non-surgical treatment	Yes
9	F	59	University	Knee	Non-surgical treatment	No
10	F	70	University	Knee	?	No
11	F	69	University	Knee	Non-surgical treatment	No
12	F	81	High school	Hip	?	No

*M* Male, *F* Female, *OS* Orthopedic surgeon, ? = Assessment outcome was unknown to the patient at time of the interview

**Fig. 1:**
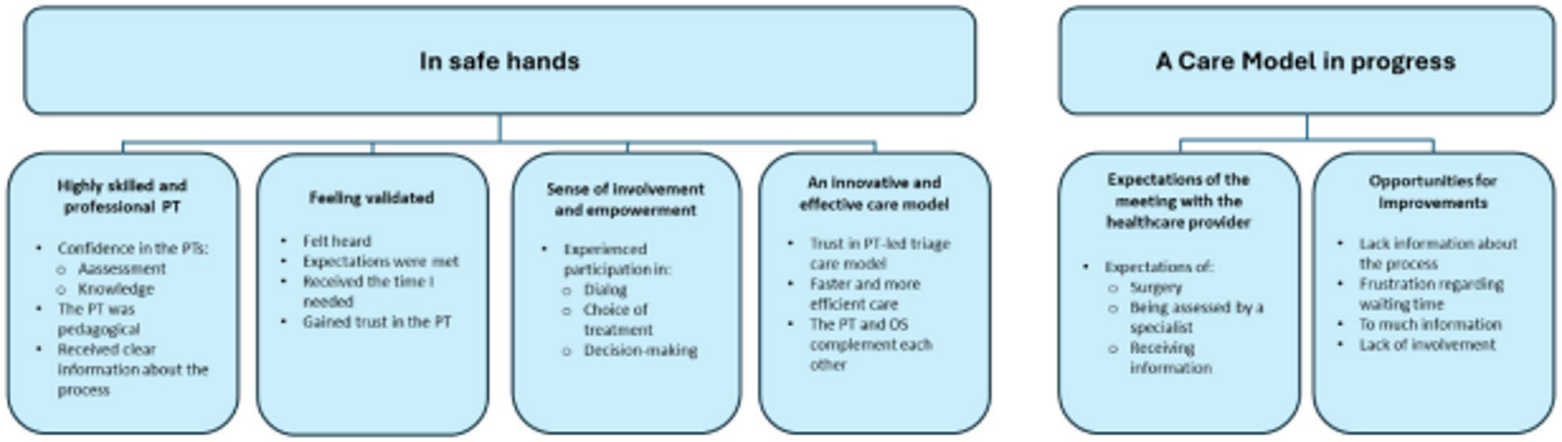
Overview of the themes, categories and subcategories

Two overarching themes were conceptualized: In safe hands and A care model in progress (Fig. 1). The theme In Safe Hands embraces patients’ positive perceptions and experiences related to various aspects of their meeting with the PT during the PT-led triage. This theme is divided into four categories. The second theme, A care model in progress, was developed based on patients’ expectations, as well as areas identified as having potential for improvement, according to the patients’ experiences with the PT-led triage assessment. This theme includes two categories (Fig. 1). Below, a description of each category and belonging subcategories are presented. An example illustrating the steps of the analysis process is presented in Table 2.

**Table 2:** Overview of analysis with examples of meaning units, codes, sub-categories, categories and themes

Meaning units	Code	Subcategory	Category	Theme
Felt like I met a good and competent person who took care of me	Felt like a competent person	Trust in the PTs knowledge	Highly skilled and professional PT	In safe hands
We talked and reasoned back and forth about the treatment	Participated in the decision about treatment	Experienced participation in choice of treatment	Sense of involvement and empowerment	
She was interested in my problems and wanted to help me to get better	The PT took an interest in my situation	Felt heard	Feeling of being validated	
Partly, this saves time by potentially reducing the need for an orthopedic visit, and additionally, the PT can also identify and filter out those who may not be as close to requiring surgery	The PT frees up resources	Faster and more efficient care	An innovative and effective care model	
Seeing a specialist, I, as a patient, expect them to take it a step further and investigate whether there might be something else, so that I receive a proper answer	Wanted to be assessed by a specialist	Expectation of being assessed by a specialist	Expectations of the meeting with the healthcare provider	A Care Model in Progress
If I could wish for something, it would be to receive a clearer and more concrete answer regarding the time frame.	Lack information about waiting times	Frustration regarding waiting time	Opportunities for Improvement	

*PT* Physical therapist, *PT-led triage* Physical therapist led triage

### Theme: in safe hands

#### Category 1: highly skilled and professional PT

##### Confidence in PTs assessment

Patients expressed that they felt that the PT conducted a thorough examination. The assessment was perceived as more comprehensive than what they had previously experienced with their primary care PT or GP. Several patients mentioned that they couldn’t think of anything more that the PT should have done.

*“It was that she conducted such a thorough examination. I felt that she didn’t miss anything. I felt that she went through absolutely everything with my knee and my hip. It didn’t seem like she missed anything*,* I felt. I felt thoroughly examined*” (participant 9).

##### Confidence in PTs knowledge

Patients perceived that they were meeting an experienced and competent professional with expertise in the field. They also expressed that the PTs explanations were reasonable, and that the PT was honest about her assessment, which contributed to their trust in the PTs knowledge.

“*When I got there*,* I knew nothing*,* but when I left*,* I felt like I knew more. And that… you could tell she knew her stuff*” (participant 12).

##### Received clear information about the process

Most of the patients reported receiving clear information about their care process. They also stated that they were informed on how to proceed with their care after the PT assessment, i.e. how the treatment pathway for patients with OA entails. This was something patients emphasized as very important. Patients, for whom surgical interventions were deemed as a possible option stated that they received information and clear instructions on how to prepare for the surgery.

*“I received a good explanation of what the process will look like moving forward”* (participant 7).

##### The PT was educational

Patients perceived that the PT explained the causes of pain and functional impairments as well as provided information about the treatment of OA in an educational and accessible manner. This contributed to the patients feeling safe and gaining increased knowledge about their condition.

“*So*,* she showed exactly how… what osteoarthritis is and what an implanted prosthesis looks like. Of course*,* it’s not as good as the original one. No*,* but she was very educational…*” (participant 4).

#### Category 2: feeling validated

##### Felt heard

All patients reported a sense of being heard. They felt that the PT listened to and understood their situation, that they were meeting a professional who was genuinely interested in their problems and wanted to help them.

“*It also puts me as a person in focus*,* and that always feels good when you have a medical symptom – to have someone who listens specifically to me and my situation. And there is a psychological value in that”* (participant 12).

##### Received the time I needed

One thing the patients were pleasantly surprised by was the length of the assessment. They felt that there was sufficient time for them, and the opportunity to get answers to their questions. The patients expressed that there was no sense of time pressure.

*“She (the PT) gave me the time it needed. I felt that there was no sense of time pressure”* (participant 2).

##### Gained trust in the PT

A consisting finding among the patients was a sense of trust in the PT. Patients stated that this trust stemmed from the PT’s calm demeanor, honesty, objectivity and knowledge. They describe feeling in safe hands during the meeting. They perceived the PT as calm and reassuring and experienced a professional and respectful encounter.

*“I felt like I was in safe hands*,* I thought it was a very good person to talk to.”* (participant 1).

##### Expectations were met

Many patients expressed that their expectations regarding the assessment with the PT were met. Some patients had expectations about surgery and felt that after the assessment by the PT they had gained hope about receiving surgical intervention.

*“I felt a sense of hope that I would get a surgery.”* (participant 12).

#### Category 3: sense of involvement and empowerment

##### Experienced participation in the dialog

Patients expressed the feeling of participation in the dialog with the PT. They had a feeling of being involved during the visit by being able to voice their concerns and ask questions.

“*She listened to what I said*,* and she understood me… oh*,* well… no*,* I thought it was good. We both got to talk”* (participant 11).

##### Experienced participation in decision-making progress

Most of the patients expressed a feeling of participation in the decision-making process about their care. However, one patient mentioned finding it hard to make the decision about surgery himself.

*“There was no one else pushing me*,* I had to make the decision myself… She was very clear about that in how she presented it. That I had to make the decision*,* and she couldn’t*,* which I think is absolutely right”* (participant 2).

”*What I found somewhat difficult was that I had to make a decision about whether to proceed with the surgery*” (participant 7).

##### Experienced participation in choice of treatment

All patients except one reported experiencing participation in the choice of treatment. Patients stated that they discussed the treatment options and associated risks with the PT, and that the PT also asked for their own treatment preference. A sense of agreement with the PT regarding the choice of treatment was described by patients for whom surgery intervention was considered suitable and not suitable.

“*She asked*,* do you want to get surgery? And I said yes*” (participant 12).

#### Category 4: an innovative and effective care model

##### Trust in the care model

The patients believed that this care model would be as effective as standard care, i.e., an assessment by an OS, and did not perceive any disadvantages in having a PT conduct the assessment. Patients expressed the PT and OS complement each other regarding their strengths and knowledge. One patient also suggested that this care model should be implemented at other hospitals.

*“I think that the care model is excellent. It truly makes full use of the expertise”* (participant 7).

##### The PT and OS are equally competent

Patients expressed thoughts about PTs and OS being equally competent regarding OA diagnosis and treatments. The patients assume that independent of the profession, they both have the necessary knowledge to make decisions regarding surgical intervention.

“*There is nothing to say that a PT could not do it as well as an OS*,* I think. So*,* I see no difference at all*” (participant 1).

##### Faster and more efficient care

Patients expressed the view that PT-led triage could provide faster and more efficient care by enabling the PT to identify those who do not require surgical interventions. This would allow patients to receive care at the appropriate level, with reduced waiting times. Patients suggested that PTs could relieve the OS from tasks within the PT’s scope, thereby freeing up surgical time for the OS.

“*This ensures that the patient receives care at the appropriate level”* (participant 10).

### Theme: a care model in progress

#### Category 1: expectations of the meeting with the healthcare provider

##### Expects to be assessed by an expert

Patients’ expectations at the meeting were to be seen by an expert in the OA field. They stated that an expert could provide answers that they had not received in primary care. Some patients also expressed a belief that an expert might identify other causes of their pain that previous healthcare providers had missed, indicating that the hospital setting creates certain expectations about meeting a specialist.

*“When seeing an expert*,* as a patient*,* I expect them to take it a step further and check if there’s something else*,* so that I can get a proper answer*” (participant 1).

##### Expectations of surgery

Some patients expressed hope for surgical intervention. Those patients wanted to be assessed and to get answers if surgical intervention would be suitable for them.

##### Expectations about receiving information

The patients that did not want surgical intervention expressed a wish to receive information about their disorder and what to do to get better. They were hoping to get a specialist opinion on how to continue their care.

“The expectation was that now, finally, I would be examined by a expert.” (participant 1).

#### Category 2: opportunities for improvements

##### Lack of information about the process

Patients expressed not receiving information about the time interval between the assessment and either the arrival of the decision letter or the meeting with an OS. They emphasized that receiving a clear timeframe for each step in the process is important, and it would make it easier to manage. One patient also mentions lacking information about the steps involved in the overall processes.

*“…but then*,* one could say that it will not happen within three months or four months*,* providing a concrete timeframe to relate to. Knowing this is very important*,* as it allows one to adjust expectations accordingly. It also makes the waiting process easier when dealing with uncertainty*,* as it provides a clearer sense of what to anticipate”* (participant 5).

##### Too much information about surgery

One patient expressed receiving excessive detailed information about the surgery. The patient mentioned knowing it is important to get all information but wished to get it later in the process and not so detailed.

*“You shouldn’t start by saying that they’re going to saw off the thigh bone*,* because I thought that was awful. I mean*,* you could… well. And of course it’s important to get concrete information and all that*,* but maybe that part should come at another stage in the process.”* (participant 5).

##### Lack of involvement in the decision-making process

As the final decisions regarding patients’ care were made during the PT–OS conference, some patients felt that they didn’t receive a clear decision about their continued care during the initial PT meeting. One patient expressed disappointment and a desire for faster communication. The same patient also felt excluded from the decision-making process, as the PT needed to consult the OS after the triage assessment, leaving the patient with little influence over the outcome.

*“She does it throughout the entire preliminary examination*,* so to speak. She carries out all the tests and everything. And he*,* the orthopedic surgeon*,* saves time because all of that is already done*,* so to say. But then… in the next step*,* I end up being left on my own.” (participant 7).*

##### Frustration regarding waiting time

All but one patient expressed frustration regarding waiting time from referral to assessment.

*“And in the situation*,* I’m in*,* it’s like… why is it taking so long? But of course*,* you know why it takes so long. Still*,* you can’t help being terribly impatient.”* (participant 2).

## Discussion

The present study found that patients with hip and knee OA who underwent PT-led triage generally had positive experiences with the care model. Patients described the feeling that they were meeting an expert in the field, participating in discussions about their care and feeling heard. They also perceived the PT-led triage care model as effective and providing faster care. However, based on the patients’ experiences, there are some aspects that need improvement, such as further involvement in the decision-making process and clear information about the process.

### Patients’ expectations

As previously reported in an RCT of the care model, patients in PT-led triage felt their expectations were met to a lesser extent compared to those who received standard care (i.e., were assessed by an OS) [14]. In contrast to this finding, the patients in the present study reported that their expectations were fulfilled. They expressed a desire to be assessed by an expert, to receive information about their OA and how to manage their OA, and some patients hoped to receive surgery intervention, and they perceived that they got what they wished for. The RCT was a quantitative study with a larger number of participants, which may explain the differences in findings. However, the present qualitative study provides a deeper understanding of patient’s experiences. The results are consistent with a systematic review of patients with low-back pain expectations and experience of healthcare, and a qualitative study of patients with musculoskeletal disorders which reports patients wanting to be assessed by a specialist and to get information on self-help to manage their pain [12, 21].

### Patients’ experiences

In the present study, patients expressed that they felt heard and involved in decision-making, including decisions regarding their continued care. This is in line with recommendations about patient-centered care (PCC). PCC is widely recognized as the core component of high-quality healthcare and is fundamental to how patients are treated and how medical care is delivered [22–25]. Beyond involving patients in decisions about their care, PCC involves elements such as empathy, respect, and caregiver engagement, as well as good communication, strong patient-provider relationship and an individualized approach [24]. Although all healthcare providers are expected to treat patients according to these principles, the extent to which this occurs may vary depending on the caregivers’ priorities and level of commitment [26]. Patients in the present study also perceived that they were treated in line with other core aspects of PCC. This supports the perception that the PT-led triage model represents a valuable approach to care.

Patients reported feeling that they were assessed by a expert and expressed trust in the PTs knowledge of OA. They also expected the caregiver at the clinic, regardless of professional title, would have the appropriate expertise to determine the best treatment option. This finding is in line with previous research, on patients’ perceptions following PT-led triage in spinal screening, which showed that patients wanted to be informed about the entire care process and wanted to be assessed by someone who made them feel respected, cared for and truly listened to them, elements that were also highlighted by patients in the present study [18]. All patients in the present study felt that they were listened to and heard. Many also reported receiving clear and adequate information about the care process. However, some patients noted a lack of information regarding the waiting time for the decision letter or the next step in the care process. This was something patients considered important and an area in need of improvement. Clear communication and timely information have previously been reported to be crucial for patients’ overall satisfaction [18], as they contribute to patients’ trust in the healthcare provider. Ensuring that patients feel confident in their caregiver’s competence is essential, as a lack of trust may lead patients to seek additional care elsewhere, thereby increasing the burden on the healthcare system and society.

One patient reported feeling excluded from the decision-making process, as the final decision regarding surgical intervention was made during the PT-OS conference. One potential way of addressing this issue could be to inform patients of the decision by phone, allowing them the opportunity to discuss the outcome and thereby feel more included process. To further optimize shared decision-making, it would be beneficial if patients could receive the decision during the PT-led triage assessment itself. At the time of the writing, the PT-led triage care model at the Sahlgrenska clinic has been revised based on the findings of the RCT [14]. To facilitate surgical decision-making during the assessment, an assigned OS is now available for immediate consultation, and if needed, a brief patient encounter during the PT-led triage. Although the new protocol has not yet been evaluated or fully implemented in the clinic, it is believed to enhance patients’ sense of involvement in the decision about their care.

Patients expressed frustration regarding the waiting times from referral to assessment at the orthopedic clinic, even though their waiting time was significantly shorter than to an assessment by an OS. This delay does not appear to be directly related to the PT-led triage care model but rather reflects broader challenges within the healthcare system. In addition to dissatisfaction with the length of the wait itself, patients emphasized the importance of receiving clear and transparent information about expected waiting times. Uncertainty about when they would be assessed contributed to feelings of frustration and reduced their sense of control. This highlights that timely information and information about waiting times function as distinct yet equally important aspects of the patient’s experience.

A large proportion of referred patients are not considered to be in need of surgical intervention [3, 4, 6] and it would be beneficial if these patients could be identified earlier in the process. One possible solution could be the use of digital triage tools upon referral, to better identify patients who are not in need of surgical consultation. Furthermore, many patients have not obtained first line treatment for OA at the time of referral to the orthopedic clinic, an essential prerequisite for consideration of surgical intervention [4, 6]. Another possible improvement would be to implement PT-led triage at primary care level. Doing so could help identify patients who have not obtained appropriate first line treatment, thereby reducing the number of unnecessary referrals to orthopedic clinics. This, in turn, could reduce waiting time and enable care to be delivered at an appropriate level within the healthcare system.

### Integration of PT-led models and advanced-scope practice in MSK care

Across international healthcare systems, there is a growing shift toward integrated MSK care models in which primary care, hospital-based services, and digitally mediated collaboration are more closely aligned [27, 28]. Timely access to PT expertise constitutes a core element of these care models. Patients’ emphasis on prompt assessment, clear communication, and continuity of care, patterns also reflected in the present study, further underscores their relevance for MSK, including OA. Within this context, PT-led triage is well positioned to support modern integrated pathways and may function as an important entry point that facilitates patient-centered navigation through the broader healthcare system. In parallel, advanced-scope PTs (APPs), who can undertake extended tasks such as advanced assessment, imaging referral, and joint injections, are increasingly incorporated into specialist MSK services to improve access and reduce bottlenecks [29, 30]. The value patients placed on timely expertise in our study is consistent with previous reports of high trust and satisfaction with APP-led care [31]. Although these roles are typically situated within specialist settings, several authors suggest that key components could be transferred to primary care with appropriate medical support, potentially reducing unnecessary referrals and improving access to MSK, and specifically OA management.

### Methodological considerations

Trustworthiness of findings in qualitative research are commonly discussed in terms of transferability, credibility and dependability [19]. Qualitative methods have historically been criticized for their limited ability to transfer the results to other settings. The study only included 12 participants, all of whom were Swedish born. As a result, the transferability of the results to a more culturally diverse population may be limited, particularly given the demographic diversity where the study was conducted. The homogeneity of the sample may also have influenced the range of perspectives captured. Patients with different cultural or linguistic backgrounds may hold different expectations of healthcare encounters, including PT-led triage, and may interpret communication, roles, and decision-making processes in ways that were not reflected in this sample. Consequently, important variations in perceptions or experiences of PT-led triage may not have been detected, which should be considered when interpreting the findings. However, the inclusion of an equal number of male and female patients, along with variation in the outcome following PT-led triage strengthens the study’s transferability. Additionally, no new information related to patients’ expectations and experiences emerged during the last interviews. Therefore, we considered data saturation to have been reached, supporting the credibility of the findings and that the research questions were answered in a sufficient thorough and reliable manner.

Triangulation was used during the entire analysis process. All interviews were analyzed independently by two authors and a third author participated in the discussion in all steps of the analysis. This also strengthens the dependability of the research findings.

When considering potential differences between interview modes, the interviewer’s ability to interpret visual and non-verbal cues varied across face-to-face, video, and telephone interviews. While face-to-face and video interviews allowed for some degree of visual triangulation, telephone interviews relied solely on verbal expression and tone. To address this, the interviewer employed consistent probing techniques across all modes and focused on ensuring clarity and depth in participants’ responses, regardless of interview format. This approach aimed to minimize mode-related variation in the interpretation of participants’ experiences.

The interviews and subsequent analysis were conducted in Swedish and translated into English as a final step. An effort was made to remain as close to the original text as possible, especially with respect to the citations. Conducting the process in Swedish strengthened the data by allowing for richer cultural and linguistic expression. However, the translation process inevitably entails a risk of losing certain cultural nuance.

The PTs doing the assessment of the patients were aware that the study was being conducted. This awareness may have influenced aspects of their clinical performance, for example by heightening their attention to communication or procedural consistency. We acknowledge this as a potential source of bias, although the PTs were instructed to provide care according to usual practice and were not involved in data collection or analysis.

PT-led triage as a care model has been reported to result in comparable or higher levels of perceived patient satisfaction compared to standard care [13, 14]. The findings of the present study support to some degree previous research in this field. However, the structure and implementation of PT-led triage may vary across settings and the results may not be directly generalized to other clinics or countries.

All authors of the present study are PTs working in primary care. Although the analysis and writing were conducted with the intention of maintaining objectivity, it cannot be ruled out that the professional background may have influenced the interpretation of the results.

## Conclusion

The main findings of this study are that patients felt they were meeting an expert, experienced a sense of involvement in their care, and viewed PT-led triage as a valuable model of care, although some patients reported a lack of information regarding the care process. The results from this study of patients’ expectations and experiences may be used to further refine and improve this model of care, thereby supporting its implementation on a broader scale.

## Supplementary Information

Supplementary Material 1.

## Data Availability

Due to General Data Protection, the interviews are not publicly available. However, they can be made available by the corresponding author in response to a reasonable request.
