# Supplementary material for: In safe hands – patients with knee and hip osteoarthritis expectations and experience of physical therapist-led triage in a secondary care setting
Source: BMC Musculoskelet Disord. 2026 Jul 7;27:584. doi: 10.1186/s12891-026-10179-3 (PMC13340124; doi:10.1186/s12891-026-10179-3)
Supplement: Supplementary file 1 — Supplementary Material 1. [file 12891_2026_10179_MOESM1_ESM.docx]

# Supplementary file

**Interview Guide**

**Personal Information**

- How old are you?
- In which country where you born?
- What is your current occupation?
- What is your educational background?

**Introductory Questions**

- Describe the reason for your referral to the orthopedic clinic
- What were your expectations before the visit?

**Experiences and Perceptions**

- How would you describe your experience during the visit to the clinic?
- How was the discussion regarding the most appropriate treatment for your osteoarthritis?
- How did you experience the decision-making process regarding your treatment in terms of your involvement?

**Positive Aspects and Areas for Improvement**

- What did you take away from your visit?
- What aspects of your visit with the physiotherapist at the orthopedic clinic did you find beneficial?
- What aspects do you think could be improved?
- The physiotherapist conducting an orthopedic assessment is a relatively new approach. Do you have any thoughts on this care model?

**Concluding Questions**

- Is there anything I have not asked that you would like to add?
- How did it feel to be interviewed?

**Follow-up Questions**

- Could you describe...
- Can you elaborate on...
- Could you clarify...
- What do you mean by that?

Reflection

______________________________________________________________________________________________________________________________________________________________________________________________________________________________________________________________________________________________________________________________________________________________________________________________________________________________________________________________________________________________________________________________________________________________________________________________
